# Protective Effects of Wine Polyphenols on Oxidative Stress and Hepatotoxicity Induced by Acrylamide in Rats

**DOI:** 10.3390/antiox11071347

**Published:** 2022-07-10

**Authors:** Roxana Banc, Daniela-Saveta Popa, Anamaria Cozma-Petruţ, Lorena Filip, Béla Kiss, Anca Fărcaş, Andras Nagy, Doina Miere, Felicia Loghin

**Affiliations:** 1 Department of Bromatology, Hygiene, Nutrition, “Iuliu Haţieganu” University of Medicine and Pharmacy, 6 Pasteur Street, 400349 Cluj-Napoca, Romania; roxana.banc@umfcluj.ro (R.B.); dmiere@umfcluj.ro (D.M.); 2 Department of Toxicology, “Iuliu Haţieganu” University of Medicine and Pharmacy, 6 Pasteur Street, 400349 Cluj-Napoca, Romania; dpopa@umfcluj.ro (D.-S.P.); kbela@umfcluj.ro (B.K.); floghin@umfcluj.ro (F.L.); 3 Department of Mathematics-Informatics, “Iuliu Haţieganu” University of Medicine and Pharmacy, 6 Pasteur Street, 400349 Cluj-Napoca, Romania; anca.farcas@umfcluj.ro; 4 Department of Veterinary Toxicology, University of Agricultural Sciences and Veterinary Medicine, 3-5 Mănăştur Street, 400372 Cluj-Napoca, Romania; andras.nagy@usamvcluj.ro

**Keywords:** polyphenols, red wine, white wine, acrylamide, hepatotoxicity, rats, oxidative stress, antioxidant activity

## Abstract

In recent years, it has been increasingly suggested that the consumption of natural polyphenols, in moderate amounts, is beneficial for health. The aim of this study was to investigate the efficacy of a red wine (the administered dose of 7 mL/kg/day being equivalent to ~16.5 mg/kg/day total polyphenols) compared to a white wine (the administered dose of 7 mL/kg/day being equivalent to ~1.7 mg/kg/day total polyphenols), on the prevention of acrylamide-induced subacute hepatic injury and oxidative stress in Wistar rats. Hepatic damage due to acrylamide intoxication (the administered dose being 250 µg/kg body weight, for 28 days, by intragastric gavage) was assessed by employing biochemical parameters (aspartate aminotransferase (AST) and alanine aminotransferase (ALT)) and by histopathological studies. Markers of oxidative damage were measured in terms of plasma malondialdehyde (MDA), hepatic Thiobarbituric Acid Reactive Substances (TBARS) and glutathione (GSH) levels, and liver antioxidant enzyme (superoxide dismutase (SOD), catalase (CAT) and glutathione peroxidase (GPx)) activities. Regarding hepatic enzyme activities, treatment with red wine significantly decreased the AST values (*p* < 0.05), while for the ALT values only a normalization tendency was observed. Treatment with red wine and white wine, respectively, significantly prevented the increase in MDA and TBARS levels (*p* < 0.05), as well as the depletion of GSH (*p* < 0.05). Red wine treatment normalized the activities of the antioxidant enzymes CAT and SOD in rats intoxicated with acrylamide, while supplementing the diet with white wine did not produce significant differences in the antioxidant enzyme activities. Histopathological findings revealed a moderate protective effect of red wine after four weeks of daily consumption. Our findings provide evidence that red wine, having a higher phenolic content than white wine, has a significant protective effect on oxidative stress and liver injury induced by acrylamide in rats, through its antioxidative activity.

## 1. Introduction

Among the alcoholic beverages widely consumed worldwide, wine remains a very popular beverage, having been consumed for hundreds of years [1,2,3]. In adults, moderate consumption of red wine is one of the typical elements of the Mediterranean diet [3,4,5,6]. The association of a moderate consumption of wine with numerous health benefits is based on its content being rich in antioxidant phenolic compounds, which have both a functional role, acting against free radicals, and a physiological role, by increasing the antioxidant capacity of the human body [1,2,4,5,6,7].

While moderate wine intake is associated with significant health benefits, excessive alcohol consumption is considered a major risk factor for many diseases (including alcoholic liver disease, cirrhosis of the liver and cancer) and deaths among adults [1,6,8]. Alcohol, consumed in excess, exerts toxic effects on the hepatocellular level and free radicals generated in excess from hepatocyte destruction cause alteration of proteins and lipids, with the consequence of intensification of oxidative stress and lipid peroxidation, and, overall, a negative effect on the cellular antioxidant defense system [9,10,11]. Therefore, oxidative stress plays a key role in the pathophysiological processes that underlie liver damage related to excessive alcohol consumption [9,10]. However, the results of several studies associate regular consumption of wine in moderate amounts with beneficial cardiovascular effects and a low incidence of deaths from atherosclerosis and coronary heart disease, effects related to increased levels of high-density lipoprotein (HDL), but which have not always been confirmed in the case of white wine [2,6,12,13,14,15]. Despite numerous studies referring to the beneficial effects of red wine, Radeka et al., have shown that white wines, not just red wines, have produced a decrease in systolic and diastolic blood pressure, total cholesterol and LDL levels, and an increase in HDL levels and serotonin and dopamine levels, following regular consumption for six weeks [16]. Moreover, moderate wine consumption (200–300 mL/day), both for red and white wine, is generally associated with a reduction in all-cause mortality, with studies highlighting some of the beneficial effects, such as regulating blood pressure, cholesterol and lipids, anti-inflammatory and anti-tumor effects, preventing diabetes, obesity, atherosclerosis, cardiovascular and neurodegenerative diseases [2,6,14,15,16]. Although chronic exposure to ethanol induces up-regulation of hepatic antioxidant enzymes in mice, the beneficial effects of red wine are thought to exceed those attributed especially to alcohol [17,18]. Therefore, this increased protection is attributed to the presence of polyphenols, powerful antioxidants, more abundant in red wines [4,18,19].

Acrylamide (ACR) is a chemical contaminant that naturally forms during heat processing, at temperatures above 120 °C, by frying, roasting, toasting, grilling or baking of foods rich in carbohydrates and low in proteins [20,21,22,23,24,25]. It is abundant in fried and roasted potato, vegetable crisps, cocoa and roasted coffee, bread and pastry, cookies, as well as in tobacco smoke, and, in lower concentrations, in breakfast cereals, biscuits, crackers, roasted nuts, homemade food, human breast milk, infant milk powder and a range of baby foods [21,23,25,26,27]. ACR can be absorbed through the digestive tract, respiratory system or through the skin and after exposure can be rapidly distributed to many tissues and organs, such as the thymus, thyroid, liver, heart, brain, spleen, kidneys, as well as the human placenta and breast milk [21,25,27,28]. It is further transformed by an enzymatic reaction involving cytochrome P450 2E1 into a more toxic and reactive epoxide, glycidamide, which is widely distributed into the tissues [21,24,26,29]. Although both ACR and glycidamide can react with proteins (e.g., haemoglobin) to form covalent adducts, only glycidamide forms covalent adducts with DNA amino groups, which cause genetic mutations and damage chromosomes [26,27,29,30]. Both Hb adducts and covalent DNA adducts of glycidamide are used as important biomarkers of AA exposure [29]. Studies have shown that ACR is neurotoxic and genotoxic and has been classified as “probably carcinogenic to humans”, carcinogenicity Group 2A, by the International Agency for Research on Cancer, based on its carcinogenicity in rodents [21,23,24,31]. In addition, in 2015, the European Food Safety Authority (EFSA) confirmed that the presence of acrylamide in foods is a public health concern, requiring continued efforts to reduce its exposure [20,29]. In this context, Regulatory Agencies have provided recommendations for reducing the formation of ACR in food, without setting mandatory limits for maximum acceptable levels of AA in food [25]. Among the recommendations made by the Spanish Agency for Consumer Affairs, Food Safety, and Nutrition (AECOSAN), in 2015, are: cooking food in the microwave or baking instead of frying, reducing the time and temperature of frying food (<175 °C) and avoiding reuse of frying oil [25]. At European Union level, “Commission Regulation (EU) 2017/2158” was issued in 2017, setting mitigation measures and benchmark levels to reduce the presence of AA in food [32].

Oxidative stress is one of the main mechanisms explaining the neurotoxicity and hepatotoxicity following ACR exposure [28,30]. Increased ROS production, as a result of ACR exposure, may adversely affect cell survival, due to cell membrane damage by oxidative degradation of lipids, proteins, and irreversible DNA modification [21,25,33]. Lipid peroxidation, estimated through the level of substances reacting with thiobarbituric acid and of hydroperoxides, as well as products of proteins’ oxidation, such as carbonylated proteins, are markers of oxidative degradation produced by ROS [33]. In addition, oxidative damage is aggravated by decreased antioxidant enzyme activities, such as those of superoxide dismutase (SOD), catalase (CAT), glutathione S-transferase (GST) and glutathione peroxidase (GPx), which act as free radical traps in conditions associated with oxidative stress [21,25,33,34]. Since both ACR and glycidamide can be conjugated to glutathione (GSH) as a detoxifying pathway of ACR, forming GSH adducts that are subsequently converted to mercapturic acids, which are used as biomarkers of ACR exposure, the consequence is GSH depletion [25,28,29]. Therefore, proteomics studies suggest that ACR-induced toxicity may be mediated by cellular oxidative stress following extensive GSH consumption [25,28]. Free radicals are produced continuously in vivo and protective antioxidant enzymes (SOD, CAT, GST, GPx), as well as reduced GSH, have roles in coping with these toxic substances [33,35].

Since it is vital to know the role of naturally occurring antioxidants in preventing the negative health effects of ACR exposure, several recent studies have evaluated these issues. Thus, Sayed et al., showed that pomegranate peel has anti-inflammatory, anti-apoptotic, and free radical scavenging activity and strong antioxidant activity that protects against ACR toxicity [21]. In another study by Hamdy et al., hesperidin and tiger nut demonstrated an antioxidant defense against ACR toxicity in breast, liver and kidney tissues [34]. Qu et al. also showed that digested jackfruit flake provides increased protection against oxidative damage caused by ACR, significantly reducing cytotoxicity and excessive ROS production in cells, thus mitigating mitochondrial disorders [36].

In order to reveal a new strategy for the prevention of oxidative stress-caused diseases, it is important to take into account that, although the health effects of phenolic compounds are supported by their powerful antioxidant activity, their influence on health will depend on the amount consumed and their bioavailability. Moreover, despite the promising potential of any compound/element/extract demonstrated in vitro, the efficacy/beneficial effects and the level of safety can only be established through in vivo toxicological studies [37,38]. As there are currently insufficient studies in chronic models to demonstrate the relationship between the bioavailability of phenolic compounds and their antioxidant effects, this experiment aimed to assess the relationship between in vitro and in vivo wine functionality, namely whether the in vivo antioxidant response of wines correlates with the in vitro antioxidant activity of wines. Thus, in vitro wine functionality was tested in a previous study, which characterized white and red wines obtained from Romanian grape cultivars. Based on the results obtained previously, the white wine and the red wine, respectively, with the richest phenolic content and the highest in vitro antioxidant activity, were selected for the present study [39].

Since the generation of ACR in food is unavoidable, a modern approach is to include functional foods in the diet, thus taking advantage of the protective effects of their biologically active components. Therefore, the present study was designed to evaluate the protective effects of wine phenolic compounds against oxidative stress and liver toxicity experimentally induced by ACR in Wistar rats. For this purpose, wine was administered to rats, in the quantity and with the periodicity that corresponded to the most frequent consumption behavior in Mediterranean countries: two to three glasses of wine with 12.5% ethanol, during a day. Thus, in this study, the wine was administered both to rats fed with a standard diet and to rats treated with ACR, in addition to the standard diet, as an experimental model for inducing oxidative stress.

## 2. Materials and Methods

### 2.1. Wine Samples and Reagents

The two wine samples used in this study were local varieties produced from old Romanian vine varieties and were purchased from a local supermarket. As we reported before, Fetească Neagră red wine (FN_Toh2010_) came from the Viticultural Region of the Muntenia and Oltenia Hills, Dealu Mare Vineyard, Tohani Wine Center, while Fetească Regală white wine (FR_Jid2011_) came from the Transylvanian Plateau Wine Region, Târnave Vineyard, Jidvei Wine Center [39]. The bottles were opened, immediately separated into tubes and stored at −80 °C.

The total phenolic content (TPC) and in vitro antioxidant activity of the wines used in this study are presented in Table 1.

Acrylamide (C_3_H_5_NO, purity > 99%) was purchased from Merck (Merck, Darmstadt, Germany) and 1,1,3,3-tetraethoxypropane (TEP) was purchased from Sigma-Aldrich (Sigma-Aldrich, Steinheim, Germany). HPLC grade reduced and oxidized glutathione were obtained from Fluka (Fluka, Buchs SG, Switzerland). Acetonitrile, methanol, formic acid, acetic acid, acetone and HPLC grade hexane were purchased from Merck (Merck, Darmstadt, Germany). All other chemicals were of analytical grade and were obtained from Merck (sodium hydroxide, hydrochloric acid, sulfuric acid, perchloric acid, 2,4-dinitrophenylhydrazine (DNPH), sodium tetraborate decahydrate, o-phthalaldehyde, acetaldehyde, TRIS hydrochloride (Tris hydroxymethyl aminomethane hydrochloride) and 1,4-dithiothreitol (DTT)).

Deionized water was obtained using a Milli-Q water purification system (Millipore, Milford, MA, USA). All other reagents used to determine the biochemical parameters were of analytical grade and were purchased from Merck and Sigma.

### 2.2. Experimental Animals

A community of 60 healthy white rats, Charles River Wistar (Crl:WI) strain, males, with an initial weight of 150 ± 14 g, was used. The animals were provided by the Center for Experimental Medicine and Practical Skills of the “Iuliu Hațieganu” University of Medicine and Pharmacy Cluj-Napoca (Romania). The rats were housed in large polypropylene cages (5 rats per cage) and kept in a controlled environment: the temperature of the animal storage room was 21 ± 1 °C, with a relative humidity of at least 60% and the daily cycle of animals was 12 h of light and 12 h of darkness, with an air change ratio of 10–20/h. Throughout the experiment, the animals had free access to the standard dry pellet diet (Cantacuzino Institute, Bucharest, Romania) and drinking water ad libitum. All procedures and treatments applied to animals in this study were performed in accordance with European Union Directive (2010/63/EU) for animal experiments and were approved by the Ethics Commission of the “Iuliu Hațieganu” University of Medicine and Pharmacy Cluj-Napoca (Romania) (protocol number 219/11.06.2014).

### 2.3. Experimental Design

The study lasted 33 days, with an animal acclimatization period of 5 days and an effective experimental period of 28 days. The animals were randomly divided into six groups, each group consisting of ten animals. All experiments on rats were conducted in the morning (in accordance with ethical principles for conducting painful experiments on animals and with the current guidelines for the care of laboratory animals). The experimental groups, the feeding regime, and the manipulations performed are presented in Table 2.

The volumes of wine or 12.5% hydroalcoholic solution administered were calculated based on a wine consumption of 500 mL/70 kg body weight/day, and an equivalent volume of 7 mL/kg/day (~16.5 mg/kg/day total polyphenols in the case of red wine) was administered. Doses were adjusted daily according to the weight of the animals. Intragastric gavage was performed once a day at the same hour using flexible feeding needles (17 Gauge, 85 mm length and 2.4 mm tip diameter) (Fine Science Tools). No samples were taken during the experiment.

The animals were observed daily for 4 weeks. It was observed whether the animals showed signs of toxicity, such as the following: pathological changes in the skin and mucous membranes, changes in appearance and fur condition, damage to other systems (respiratory system, central nervous system) or changes in behavior and general condition.

### 2.4. Collection of Biological Material

After 4 weeks, the rats were deprived of food for 12 h, and blood samples were collected, in tubes treated with ethylenediaminetetraacetic acid (EDTA), from the retro-orbital venous sinus, after instillation of the eye drops with local anesthetic, oxybuprocaine 0.4% (Benoxi 4 mg/mL). Plasma was separated by centrifugation for 15 min at 1600× *g* and 4 °C and was used immediately to determine reduced and total GSH. The rest of the plasma samples were frozen in liquid nitrogen and stored at −80 °C for further analysis (determination of total plasma level of malondialdehyde (MDA) and determination of transaminases).

The animals were sacrificed by dislocating the cervical spine under isoflurane general anesthesia and the liver was taken and weighed. Liver tissue samples were taken immediately, some being fixed in 10% formalin solution, buffered at neutral pH, for incorporation into paraffin, while others were washed three times in 0.9% ice-cold saline solution to remove blood, wiped individually on filter paper and quickly frozen in liquid nitrogen and stored at −80 °C, being subsequently used for the preparation of liver homogenates.

### 2.5. Histopathological Evaluation of Liver Tissue

For the histological exam, the hepatic samples were fixed in 10% buffered neutral formalin and embedded in paraffin. The sections were made with a high-precision microtome Leica RM 2125 RT, at 5-µm thick, and stained by the hematoxylin–eosin (HE) and Sirius red (SR) methods. The slides were examined under a BX51 Olympus microscope, Olympus DP 25 digital camera and processed using the Olympus Cell B image acquisition and processing program.

### 2.6. Evaluation of Liver Function

Transaminases, aspartate aminotransferase (AST) and alanine aminotransferase (ALT), which are useful markers for assessing the level of liver cell damage, were determined in plasma samples by spectrophotometric tests using commercial kits provided by Randox Laboratories (Ardmore, Nothern Ireland, Great Britain).

### 2.7. Preparation of Hepatic Tissue Homogenates

Each pre-weighed frozen liver tissue was homogenized on an ice bath for 10 min in a Polytron PT 1200 E homogenizer with 10 volumes of 50 mM Tris buffer containing 10 mM EDTA (pH 7.5). The homogenate was centrifuged for 10 min at 1000× *g* and 4 °C to remove all cellular debris and the resulting supernatant was used to determine oxidative stress parameters (Thiobarbituric Acid Reactive Substances (TBARS), GSH) and antioxidant enzymes (SOD, CAT and GPx). The expression of the parameters determined in the supernatant was done per mg of protein.

### 2.8. Protein Quantification in Liver Tissue Homogenates

The protein content in liver homogenates was quantified in the supernatant by the method described by Bradford [40]. This method is based on the color reaction of proteins with an acidic solution of Coomassie Brilliant Blue G-250, called Bradford reagent. Samples of tissue homogenate were diluted so that their protein content was 5–100 μg protein/100 μL homogenate. A volume of up to 0.1 mL (adjusted to 0.1 mL with phosphate buffered saline) was taken from this diluted tissue homogenate, to which 5 mL of Bradford reagent were added. After 5 min, the absorbance was read at 595 nm against the blank (prepared from 0.1 mL of buffer and 5 mL of Bradford reagent) and the protein content (expressed in mg/mL) was determined from the calibration curve.

### 2.9. Evaluation of Oxidative Stress by Means of Biomarkers Determined in Plasma

#### 2.9.1. Determination of Lipid Peroxidation by Quantification of Malondialdehyde in Rat Plasma

The degree of lipid peroxidation was assessed by quantitative analysis of MDA using a Waters Acquity Ultra Performance Liquid Chromatography (UPLC) system coupled with a Waters Acquity PDA (Waters, Milford, USA). For this purpose, 75 μL rat plasma sample were used. The sample preparation consisted in a hydrolysis step at 60 °C in a waterbath, in the presence of 25 μL 6M NaOH, followed by protein precipitation with 63 μL 35% perchloric acid and derivatization with 100 μL 5mM 2,4-dinitrophenylhydrazine (in 2N HCl). The final derivatization product was extracted in 1.2 mL n-hexane, followed by the evaporation of the organic layer under a stream of nitrogen. The residue was dissolved in 100 μL mobile phase (a mixture of 1% formic acid/acetonitrile, 62/38, *v*/*v*) and subjected to UPLC-PDA analysis.

Chromatographic separation was achieved on a BEH C18 column (50 mm × 2.1 mm i.d., 1.7 µm) from Waters (Waters, Milford, USA) preceded by a 0.2 µm online filter. A 7.5 min gradient elution was performed with a mixture of 1% formic acid/acetonitrile as the mobile phase. The flow rate was 0.3 mL/min and the absorbance of the eluent was monitored at 301 nm [41,42].

#### 2.9.2. Determination of Reduced and Total Glutathione Plasma Levels

Reduced and total GSH were determined in plasma samples by UPLC with fluorescence detection. A Waters Acquity UPLC system coupled with a Waters Acquity Fluorescence detector was used (Waters, Milford, CT, USA).

For the quantification of reduced GSH, 100 μL of rat plasma samples were needed. The sample preparation involved a simple protein precipitation step with sulfosalicylic acid, followed by derivatization for 1 min with ortho-phtalaldehyde.

In case of the total GSH, an additional step was needed. After deproteinization, the oxidized GSH was reduced by incubation with 100 mM DTT, for 5 min at room temperature.

Chromatographic separation was carried out using a BEH C18 column (100 mm × 2.1 mm i.d., 1.7 µm) from Waters (Waters, Milford, CT, USA) preceded by a 0.2 µm online filter. For reduced GSH, isocratic elution was performed at a flow rate of 0.3 mL/min, with a mixture of methanol and 0.25% acetic acid (pH 6.9, with 6 M NaOH) as the mobile phase. In the case of the total GSH, the same mixture was used as the mobile phase, but a gradient elution was needed. The selected excitation and emission wavelengths were 350 nm and 420 nm, respectively [42,43].

### 2.10. Evaluation of Oxidative Stress in Liver Tissue

#### 2.10.1. Determination of Lipid Peroxidation by Quantifying TBARS in Liver Tissue

Liver tissue lipid peroxidation was measured by the fluorimetric method for the determination of TBARS, described by Conti et al. [44]. This method depends on the formation of MDA, as the final product of lipid peroxidation, which reacts with thiobarbituric acid and forms a fluorescent adduct, which can be measured spectrofluorimetrically at 534 nm. The TBARS method is nonspecific for MDA, fatty peroxide-derived decomposition products other than MDA being thiobarbituric acid positive. For the quantification, 50 μL of tissue homogenate were mixed with 1 mL of 10 mM solution of 2-thiobarbituric acid in 75 mM solution of K_2_HPO_4_ (pH 3.0), followed by stirring for 5 s, then the mixture was heated in a water bath at 95 °C for an hour. After sudden cooling on ice, the reaction product was extracted into 5 mL of n-butanol. Its concentration was determined in the organic phase, after separation by centrifugation for 10 min at 1500× *g* and 4 °C. Emission intensity measurement was performed at 534 nm with a Perkin Elmer LS 45 spectrofluorimeter (Norwalk, CT, USA), using a synchronous fluorescence technique at a wavelength difference between excitation and emission (Δλ) of 14 nm. The concentrations were expressed in nmol/mg protein.

#### 2.10.2. Determination of Reduced Glutathione Levels in Liver Tissue

The fluorimetric method described by Hu [45] was used to measure reduced GSH levels in liver tissue. This method of quantifying GSH by fluorescence is based on the reaction of GSH with o-phthalaldehyde, resulting in the formation of a fluorescent product that can be measured spectrofluorimetrically. Briefly, a volume of 0.5 mL tissue homogenate was added to 0.5 mL of 10% (*m*/*v*) trichloroacetic acid solution. After being kept on ice for 10 min, the mixture was centrifuged for 10 min at 3000× *g* and 4 °C and 0.2 mL of supernatant were mixed with 1.7 mL of phosphate buffer and 0.1 mL of o-phthalaldehyde. After 15 min, the emission intensity was measured at 420 nm, at an excitation of 350 nm, against a blank containing deionized water, instead of the tissue homogenate, using a Perkin Elmer LS 45 spectrofluorimeter (Norwalk, CT, USA). Concentrations were expressed in nmol/mg protein.

#### 2.10.3. Determination of Antioxidant Enzymes in Liver Tissue

The determination of superoxide dismutase activity was performed by the cytochrome C reduction test, described by Flohé and Otting [46]. Superoxide dismutase catalyzes the dismutation of the superoxide radical (O_2_^−^•) in hydrogen peroxide (H_2_O_2_) and oxygen (O_2_), thus being a defense agent against the toxic effects of superoxide. The superoxide radical (O_2_^−^•) is generated by the xanthine-xanthine oxidase system in the presence of oxygen. O_2_^−^• reacts with ferricytochrome C, which can be continuously monitored by recording the absorbance at 550 nm. In the presence of SOD, the reduction of cytochrome C is inhibited, due to the decrease in the concentration of superoxide ions. The SOD concentration in the sample could, thus, be calculated from the degree of inhibition of cytochrome C reduction, using a calibration curve obtained using SOD standards of known concentrations. Absorbance measurements were recorded with a Jasco V 530 UV-Vis spectrophotometer (Tokyo, Japan).

A unit (U) of SOD activity was defined as the amount of enzyme capable of inhibiting by 50% the rate of reduction of cytochrome C under the conditions specified. The results were expressed in U SOD/g protein.

The determination of catalase activity was performed by the method described by Pippenger et al. [47]. Catalase is considered to be an enzyme with an antioxidant role because it regulates the level of H_2_O_2_ which, in its absence, could increase and lead to the appearance of an excess of highly reactive •OH radicals. The method consisted of measuring the change of the absorbance of a solution of 10 mM H_2_O_2_ in 0.05 M phosphate buffer (pH 7.4) at 240 nm. A unit of enzymatic activity was defined as the amount of catalase that induced a reduction in absorbance of 0.43 times over a period of 3 min, at 25 °C. The activity was expressed in U/mg protein, being calculated according to the following formula:

CAT = A_240_/0.43 × 0.02 (mg/mL),

where A_240_ is the absorbance at 240 nm. Absorbance changes were monitored using the Jasco V 530 UV-Vis spectrophotometer (Tokyo, Japan).

The determination of glutathione peroxidase activity was performed by an indirect method, described by Flohé and Günzler [48]. GPx is a selenoprotein that catalyzes the reaction between a hydroperoxide (e.g., H_2_O_2_) and GSH, as an electron donor, leading to the formation of oxidized glutathione (GSSG) and water. The method used was based on monitoring the decrease in NADPH concentration, in the presence of which GSSG formed in the reaction was converted to GSH by glutathione reductase (GSSG-R). GSSG formed during the GPx reaction was reduced instantly and continuously by an excess activity of glutathione reductase, ensuring a constant level of GSH. Concomitant oxidation of NADPH was monitored photometrically. The reduction of absorption to 340 (or 365) nm was monitored for 6 min, using the Jasco V 530 UV-Vis spectrophotometer (Tokyo, Japan). The non-enzymatic reaction rate was assessed by replacing the test sample with buffer.

The activity of the enzyme was defined as the amount of GPx which induced a net decrease in GSH of 10% of the initial concentration, in one minute, at 37 °C and at pH 7. The calculation took into account the stoichiometry of the reaction and the molar extinction coefficient of NADPH. Thus,

A = 0.868 (Δ[NADPH]/[GSH]_0_t)(Vi/Vs),

where [NADPH] is the molar concentration of NADPH, [GSH]_0_-the initial concentration of GSH, t-duration of reaction, Vi-the volume of incubation mixture, and Vs-the volume of the sample to be analyzed. Activity was reported at 1 mg protein for liver tissue homogenates.

### 2.11. Statistical Analysis

Statistical analysis of data was performed using the SPSS statistical program (version 13.0). All data were expressed as mean ± SEM (standard error of the mean). To evaluate the data variations, the analysis of the unifactorial variance (One-Way ANOVA) was used, followed by the Bonferoni test as a post-test for multiple comparisons. The level of statistical significance was set for a value *p* ≤ 0.05.

## 3. Results

### 3.1. General Toxicity: Evolution of Animal Body Weight, Absolute and Relative Weight of the Liver

All animals survived throughout the experimental period. There were no changes in the external appearance of the animals, but in terms of their behavior, a state of agitation and hypermotility of the animals in the groups treated with ACR (PC, WW + ACR, RW + ACR) was observed from the 17th experimental day, compared to those who were not given ACR (C, WW, RW).

**Figure 1:**
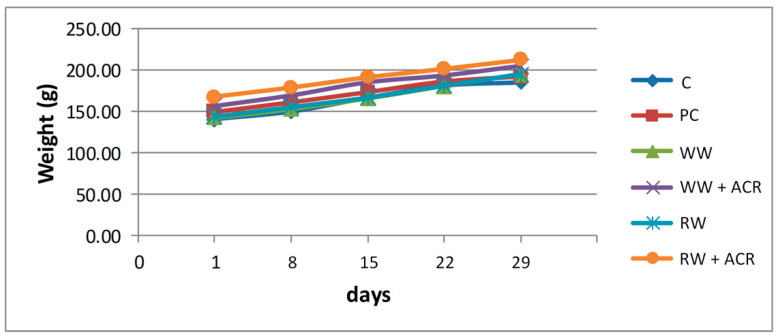
The evolution of the average body weight of the animals from the 6 groups, during the entire experimental period. C−hydroalcoholic solution group; PC−hydroalcoholic solution + acrylamide group; WW−white wine group; WW + ACR−white wine + acrylamide group; RW−red wine group; RW + ACR−red wine + acrylamide group.

The results regarding the evolution of body weight, at the end of the 28 day experimental period, for the 6 groups of animals included in the study, are presented in Figure 1. The initial body weight and the final body weight of the rats from the 6 experimental groups are presented in Supplementary Table S1.

At the end of the 4 experimental weeks, the final body weight differed significantly only for rats in the RW + ACR group, not for those in the WW + ACR group, when compared to the PC group. No significant differences were observed in terms of weight gain in the animals in the WW + ACR group and the RW + ACR group, respectively, when compared to the PC group, nor in the animals in the WW group and the RW group, respectively, when compared to the control group.

Table 3 shows the absolute and relative liver weight of rats in each experimental group.

At the end of the experimental period, the relative liver weight of rats in the PC group was significantly higher (*p* < 0.05) than that of the rats in the C group. Also, the relative liver weight was significantly increased (*p* < 0.05) in the WW + ACR group compared to the C group. In contrast, red wine supplementation in the RW + ACR group prevented a significant increase in the relative liver weight of rats compared to the C group. No significant differences in relative liver weight were obtained between the WW + ACR and RW + ACR groups, respectively, and the PC group.

### 3.2. Histopathological Examination of Rat Liver

**Figure 2:**
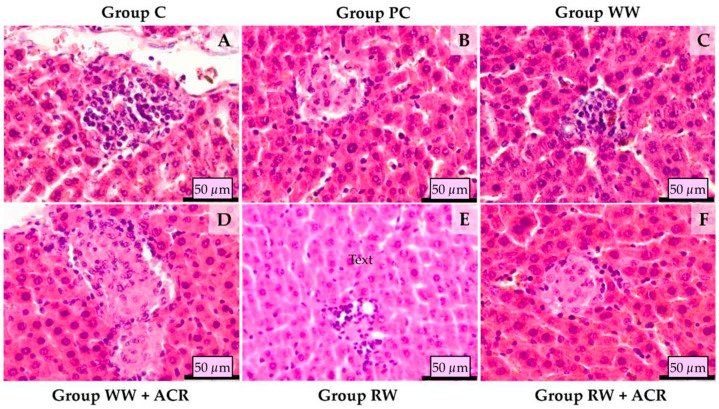
Hepatic histology in rats from different experimental groups (HE stain). Control group treated with 12.5% hydroalcoholic solution (**A**), showing perivascular lymphohistiocytic inflammatory infiltrate; Positive control group treated with 12.5% hydroalcoholic solution and acrylamide (**B**), presenting a focus of hepatic fibrosis and lymphohistiocytic inflammatory infiltrate; Group treated with Fetească Regală white wine (**C**), showing focal lymphohistiocytic infiltrate; Group treated with Fetească Regală white wine and acrylamide (**D**), showing large focus of hepatic fibrosis and lymphohistiocytic inflammatory infiltrate; Group treated with Fetească Neagră red wine (**E**), showing minimal lymphohistiocytic inflammatory cell infiltrate in the portal space; Group treated with Fetească Neagră red wine and acrylamide (**F**), presenting focus of fibrosis, minimal lymphohistiocytic inflammatory infiltrate.

Histological studies of the liver of rats from the 6 experimental groups are illustrated in Figure 2.

Histological examination of the liver samples was performed on HE and SR stained slides. Liver sections from the control group (Group C) treated with 12.5% hydroalcoholic solution showed minimal lymphohistiocytic inflammatory infiltrate around the portal spaces, as well as minimal perivascular collagen deposition in the same areas. Animals from the positive control group (Group PC) treated with 12.5% hydroalcoholic solution and ACR presented several foci of hepatic fibrosis and lymphohistiocytic inflammatory infiltrate, as well as minimal perisinusoidal collagen deposition. The hepatic sections in rats from the group treated with Fetească Regală white wine (Group WW), presented rare foci of lymphohistiocytic infiltrate and minimal collagen deposition around the portal spaces. Histological examination of the hepatic sections from the group treated with Fetească Regală white wine and ACR (Group WW + ACR) showed minimal lymphohistiocytic infiltration in the portal areas, and several foci of lymphohistiocytic infiltrate and small foci of hepatic fibrosis throughout the parenchyma. The sections from the animals of the group treated with Fetească Neagră red wine (Group RW) showed normal histology, the only modification observed was a minimal mononuclear inflammatory cell infiltrate in the portal areas. The hepatic sections from the group treated with Fetească Neagră red wine and ACR (Group RW + ACR) showed mild to severe lymphohistiocytic inflammatory infiltrate around portal spaces. In the parenchyma scattered foci of fibrosis with minimal lymphohistiocytic inflammatory infiltrate was observed.

### 3.3. Biochemical Parameters of Liver Damage

The effects of intragastric administration of white wine and red wine, respectively, on the plasma levels of AST and ALT enzymes, are presented for all experimental groups in Table 4.

The plasma levels of AST in the PC group, treated with ACR, were significantly increased (*p* < 0.05) compared to those of group C, which received only 12.5% (*v*/*v*) hydroalcoholic solution. Also, ACR treatment led to an increase in the plasma level of ALT in group PC compared to group C (*p* < 0.05). Supplementation with red wine in the RW + ACR group significantly prevented the increase of the AST level compared to the PC group, while observing a trend of normalization of the ALT value, statistically insignificant compared to the PC group. For the groups treated only with white wine and red wine, respectively, no significant differences in plasma levels of AST and ALT were obtained compared to the control group.

### 3.4. Effects of Wine Polyphenols on Acrylamide-Induced Oxidative Stress Assessed by Plasma Concentration of Biomarkers

#### 3.4.1. Determination of Lipid Peroxidation by Quantification of Malondialdehyde in Rat Plasma

**Figure 3:**
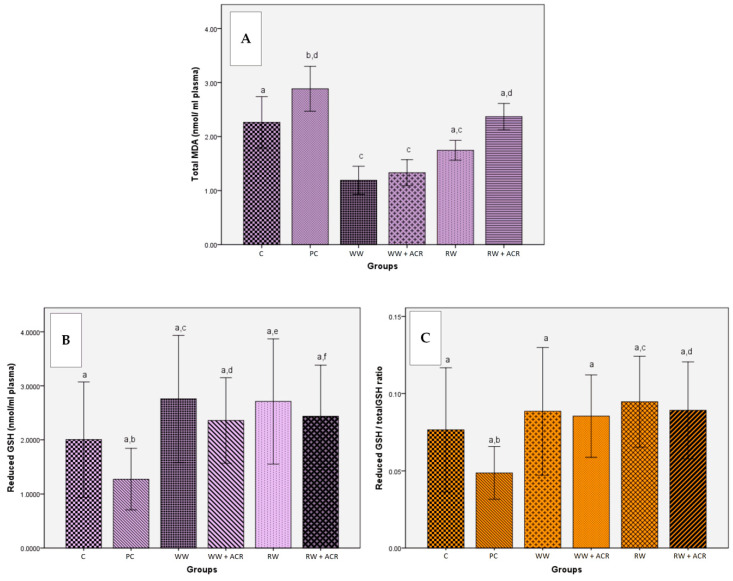
Effects of white/red wine on: total MDA plasma level (nmol/mL) (**A**), reduced GSH plasma level (nmol/mL) (**B**), and the reduced GSH/total GSH ratio (**C**) in rats from ACR-treated groups compared to untreated ones (*n* = 10). Bars marked with the same letter do not differ significantly (*p* < 0.05). C−hydroalcoholic solution group; PC−hydroalcoholic solution + acrylamide group; WW−white wine group; WW + ACR−white wine + acrylamide group; RW−red wine group; RW + ACR−red wine + acrylamide group.

The influence of intragastric administration of white wine and red wine, respectively, on the total level of MDA in the plasma of the animals from the 6 experimental groups is presented in Figure 3A. A representative chromatogram for the determination of the total MDA level in a rat plasma sample is shown in Supplementary Figure S1.

ACR treatment led to an increase in plasma MDA in the PC group compared to the control group (*p* < 0.05). The MDA value was lower in both the WW + ACR group and the RW + ACR group, compared to the PC group, but the MDA reduction was only statistically significant in the case of the first group, WW + ACR (*p* < 0.05). The groups that received one of the 2 wine samples `in addition to the standard diet, without inducing their oxidative stress with ACR, showed a low plasma level of MDA compared to the control group, with significant differences between the WW and C groups (*p* < 0.05), and without significant differences between RW and C.

#### 3.4.2. Determination of Reduced and Total Glutathione Plasma Levels

The effects of intragastric administration of white wine and red wine, respectively, on the plasma levels of reduced GSH and on the ratio of reduced GSH/total GSH are presented for all experimental groups in Figure 3B and Figure 3C, respectively. A representative chromatogram of total GSH and reduced GSH, respectively, from a rat plasma sample are shown in Supplementary Figure S2 and S3, respectively.

The reduced GSH plasma level in the PC group, treated with ACR, was not statistically different compared to that of the control group, which received only 12.5% (*v*/*v*) hydroalcoholic solution. Wine supplementation significantly prevented the reduction of the reduced GSH level in the WW + ACR and RW + ACR groups (*p* < 0.05) compared to the PC group. Treatment of animals in the PC group with ACR did not produce a significant decrease in the reduced GSH/total GSH ratio compared to the control group. Wine supplementation in ACR-treated groups only significantly prevented the reduction of the reduced GSH/total GSH ratio in the RW + ACR group (*p* < 0.05), compared to the PC group. For the groups treated only with white wine and red wine, respectively, no significant differences, in terms of the reduced GSH plasma levels or in the reduced GSH/total GSH ratio, were obtained, compared to the control group.

### 3.5. Effects of Wine Polyphenols on Acrylamide-Induced Oxidative Stress in Liver Tissue

#### 3.5.1. Effects of Wine Polyphenols on Lipid Peroxidation

**Figure 4:**
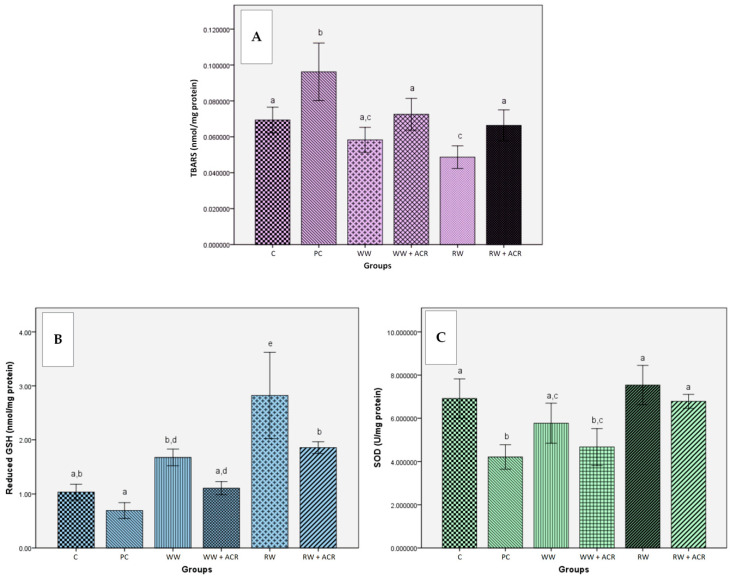

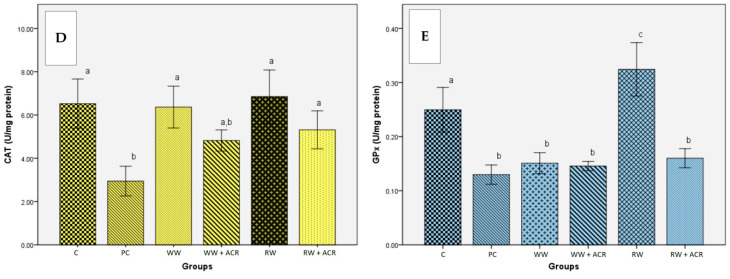
Effects of white/red wine on hepatic TBARS level (nmol/mg protein) (**A**) and reduced GSH hepatic level (nmol/mg protein) (**B**) in rats from ACR-treated groups compared to untreated ones (*n* = 10). Effects of white/red wine on superoxide dismutase activity (U/mg protein) (**C**), catalase activity (U/mg protein) (**D**), and glutathione peroxidase activity (U/mg protein) (**E**) in liver tissue of rats from ACR-treated groups compared to untreated ones (*n* = 10). Bars marked with the same letter do not differ significantly (*p* < 0.05). C−hydroalcoholic solution group; PC−hydroalcoholic solution + acrylamide group; WW−white wine group; WW + ACR−white wine + acrylamide group; RW−red wine group; RW + ACR−red wine + acrylamide group.

The influence of intragastric administration of white wine and red wine, respectively, on the hepatic level of TBARS, for the experimental groups included in the study, is presented in Figure 4A.

As can be seen from Figure 4A, hepatic TBARS levels increased significantly in the PC group treated with ACR compared to the control group (*p* < 0.05), while supplementation with wine in the groups WW + ACR and RW + ACR significantly prevented (*p* < 0.05) the increase in TBARS level compared to the PC group. For the groups treated only with white wine, or with red wine, respectively, the decrease of the TBARS value was only significant in the case of the RW group (*p* < 0.05) compared to the control group.

#### 3.5.2. Effects of Wine Polyphenols on Reduced Glutathione Hepatic Levels

The effects of intragastric administration of white wine and red wine, respectively, on the hepatic level of reduced GSH are shown in Figure 4B.

The hepatic level of reduced GSH in the PC group, treated with ACR, was not statistically different from the control group, which received only 12.5% (*v*/*v*) hydroalcoholic solution. In the case of the RW + ACR group, a significantly increased hepatic level of GSH was obtained (*p* < 0.05) compared to the PC group, while between the WW + ACR and PC groups the differences were not statistically significant. In the groups treated only with white wine and red wine, respectively, the hepatic level of reduced GSH was significantly increased for the groups WW and RW (*p* < 0.05) compared to the control group.

#### 3.5.3. Effects of Wine Polyphenols on Liver Antioxidant Enzymes Activities

The results of the in vivo study regarding the influence of wine samples on the activity of the antioxidant enzymes SOD, CAT and GPx in the liver are presented in Figure 4C–E.

A significant decrease (*p* < 0.05) in SOD activity was observed in rats from the PC group, treated with ACR, compared to those in the control group, which received only 12.5% (*v*/*v*) hydroalcoholic solution. Wine intragastric administration in groups of rats with ACR-induced oxidative stress led to a significant increase in SOD activity only for the RW + ACR group (*p* < 0.05) compared to the PC group. In the WW and RW groups, treated only with wine, there were no significant differences in SOD activity compared to the control group.

A significant reduction (*p* < 0.05) in the level of CAT activity was evident for the PC group compared to the control group. For the RW + ACR group, a significantly increased value (*p* < 0.05) of CAT activity was obtained, compared to the PC group. No significant differences were observed between the WW and RW groups, respectively, and the control group in terms of CAT activity levels.

GPx activity was significantly reduced (*p* < 0.05) in the animals from the PC group versus the animals from the control group. No significant differences were observed between the WW + ACR and RW + ACR groups, respectively, and the PC group in terms of GPx activity levels. Wine intragastric administration for the RW group has led to a significant increase in GPx activity (*p* < 0.05) compared to the control group and to a decrease in enzyme activity for the WW group.

## 4. Discussion

The use of ACR as an inducer of oxidative stress in this study was based on the fact that, although ACR poses a significant risk to human health, its widespread presence has been found in a range of processed foods, leading to an estimate of dietary exposure of 0.4 µg ACR/kg body weight/day [49,50]. Since Yousef and El-Demerdash [51], in their study, reported disorders of oxidative status and enzymatic activities produced by ACR in rats, the effects being pronounced at high doses, we chose to use the minimum dose of ACR (250 µg/kg body weight), at which these authors observed clinical signs of general toxicity and a risk of organ damage.

Numerous studies have suggested that regular and moderate wine consumption can reduce oxidative stress and inflammation, leading to a reduction in the incidence of coronary heart disease [2,6,52], these effects being attributed to phenolic compounds present in wines. In order to evaluate the hypothesis that the effects of wines on oxidative stress are dependent on their polyphenolic content and on their in vitro antioxidant activity, the diet of rats was supplemented with two different wine samples, white and red, respectively, and the oxidative stress conditions were induced by ACR-treatment. As shown above (Table 1), the two wine samples chosen for this study were the most representative, having the richest phenolic content and the highest in vitro antioxidant activity, among red and white wines, respectively, characterized in our previous study [39]. In comparison, the two wine samples showed significant differences in terms of total and individual content of phenolic compounds and in terms of in vitro antioxidant activity.

Although the intake of phenolic compounds associated with red wine (~16.5 mg total polyphenols/kg body weight/day) in the diet of animals from the RW + ACR group was much higher than that associated with white wine (~1.7 mg total polyphenols/kg body weight/day) to animals in the WW + ACR group, after 4 weeks neither of the two wines contributed to increased weight gain, compared to rats from the PC group.

Hepatotoxicity caused by ACR led to an increase in liver size in groups treated with ACR. This enlargement of the liver may have been due to hepatocyte proliferation as a result of chemically-induced hyperplasia, due to ACR and alcohol, or to an increase in the volume of individual hepatocytes. These results are explained by the fact that both ACR and alcohol are enzyme inducers, primarily of CYP2E1, but also of GST, which can lead to increased liver weight [53,54,55,56]. Regarding the absolute and relative weight of the liver, the intragastric administration of white wine and red wine, respectively, did not significantly protect against increase in liver weight, caused by ACR, in the WW + ACR and RW + ACR groups, respectively, compared to the PC group.

The results obtained suggest that the concomitant administration of wine samples to ACR-treated rats did not result in a change in relative and absolute liver weights in the direction of the values obtained for the control group.

The histopathological examination aimed at identifying morphological changes in the liver (considered the possible target organ) following the administration of alcohol in a concentration of 12.5% (in the form of hydroalcoholic solution, with either white wine or red wine), or 12.5% alcohol associated with acrylamide, respectively.

The histopathological study of the liver for the control group revealed histo-architectonic features similar to that of the groups that received white wine and red wine, respectively. The morphological changes in these 3 groups were discrete, following subacute alcohol consumption. The images obtained following the histopathological evaluation in the animals from the groups treated with ACR showed slight morphological changes, the differences observed between the 3 groups being minor. Thus, the most affected were the PC and WW + ACR groups, which showed micro-foci of hepatocyte lysis, lymphohistiocytic infiltrate in the portal spaces and minimal collagen deposition. In the ACR-treated group, whose diet was supplemented with red wine, the severe lymphohistiocytic inflammatory infiltrate in the portal spaces suggests that the administration of red wine failed to visibly protect the liver from the effects of subacute intoxication with low doses of ACR. In contrast, collagen deposition, was lower in the RW + ACR group compared to the PC group. This proliferation of collagen fibers in ACR-treated groups could have been a result of hepatic oxidative stress which, according to Li et al. [52], stimulates collagen synthesis.

A less obvious hepatic protective effect of red wine may be the consequence of the experimental study period of four weeks being of too short a duration, such that the administration of wine was subacute, especially given that the hepatic protective effects of polyphenols have been reported following longer-term consumption of red wine. Thus, Uzma et al. [57] achieved a significant attenuation of carbon tetrachloride-induced liver damage in rats by supplementing the diet with red wine for 8 weeks.

The hepatocellular cytoplasmic enzymes AST and ALT serve as indicators of liver function and integrity, being released into the systemic circulation in the case of liver damage, such as hepatocellular necrosis, with cell membrane degradation [21,53,58]. The activities of these enzymes are usually increased in acute hepatotoxicity or mild hepatocellular damage but tend to decrease with prolonged intoxication due to liver damage [58]. In our study, the evolution of ALT and AST values showed significant increases (*p* < 0.05) of the activities of these enzymes in the PC group compared to the control group, suggesting hepatocyte membrane permeability and ALT and AST migration in the intercellular space. Permeabilization of the hepatocyte membrane may have occurred as a consequence of the damage caused by the binding of acrylamide or metabolites, such as glycidamide, to membrane proteins [21]. The values obtained for AST showed a significant decrease (*p* < 0.05) in the case of the ACR-treated group that received additional red wine, compared to the PC group. The results obtained for AST indicated that red wine polyphenols had a protective effect against the hepatotoxicity of acrylamide in the RW + ACR group. This protection of red wine was not fully confirmed in the case of the values obtained for ALT in the RW + ACR group, compared to the PC group, In this case only a trend of normalization of ALT values in the direction of the values obtained for the control group was observed. The dynamics of transaminases values were consistent with data published by other authors only in case of the AST. The lower hepatoprotective effect may have been due either to supplementing the diet with wine for too short a time, the effects of polyphenols being visible only after chronic wine administration, or to an insufficient polyphenolic intake to ensure liver protection, even in the case of red wine. Thus, Alturfan et al. [49] reported significantly low levels of transaminases following dietary supplementation of rats with resveratrol, although most likely the hepatic protective effect was due to the administration of resveratrol in high doses (30 mg/kg body weight/day).

Oxidative stress is a phenomenon caused by an imbalance between the production and accumulation of ROS in cells and tissues and the antioxidant defense capacity, resulting in the oxidative damage of important cellular macromolecules, such as lipids, DNA and proteins [33,59,60,61]. Besides DNA damage, oxidative stress mediated by ROS may result in lipid peroxidation [33,59]. For instance, an excess of hydroxyl radical and peroxynitrite can cause lipid peroxidation, thus damaging cell membranes and lipoproteins [59]. Among several available markers of lipid oxidation, MDA, a volatile β-scission product formed from the peroxidation of polyunsaturated fatty acids is very popular. Since MDA is toxic and mutagenic, it is one of the most studied products of peroxidative damage [33,59].

The data obtained in this study showed that ACR produced an increase in lipid peroxidation, expressed by an increase in plasma MDA levels. The decrease in plasma MDA concentrations observed in the study in the WW + ACR and RW + ACR groups suggests that the whole lipid peroxidation process was diminished by regular supplementation of the wine diet. Our data are consistent with those obtained by Montilla et al. [62], who reported a significant reduction in lipid peroxidation, assessed by the MDA level determined in the plasma of rats with induced oxidative stress, due to dietary supplementation with red wine. In contrast to these results, Macedo et al. [63] did not obtain significant differences in plasma MDA levels after administration of 3 samples of red wine, having low, medium and high in vitro antioxidant activity.

GSH is the main cellular antioxidant and one of the most important parameters for assessing oxidative damage [25,33]. The antioxidant effects of wines were demonstrated by the results obtained for the two parameters, reduced GSH and reduced GSH/total GSH ratio, in animal plasma. Thus, a significant increase (*p* < 0.05) in the reduced GSH plasma level was evident in both the WW + ACR group and the RW + ACR group compared to the PC group, while the reduced GSH/total GSH ratio was significantly increased (*p* < 0.05) only in the RW + ACR group. This showed that wine ingestion resulted in a protective effect, due to the phenolic antioxidants present in the two types of wine, but the protective effect was significantly more pronounced for the red wine, which has a higher phenolic content than the white wine. Similar results were reported by Montilla et al. [62], whose study on rats with induced oxidative stress obtained a significant increase in plasma GSH following the administration of red wine.

In this study, plasma levels of reduced GSH decreased while MDA levels increased in ACR-treated rats. The increase in MDA levels, the indicator marker of the degree of lipid peroxidation, was in agreement with the findings of Filipovic et al. [25], which suggested that intensified lipid peroxidation is a consequence of GSH depletion as a result of oxidative stress.

The generation of ROS in rat liver was found to be significantly increased (*p* < 0.05) in the PC group treated with ACR compared to C, WW and RW groups, which did not receive ACR. These results confirmed the harmfulness of ACR in producing an oxidative stress state, with changes of all the following evaluated oxidative stress markers being observed: TBARS, GSH and the activities of antioxidant enzymes (SOD, GPx and CAT).

ACR is able to interact with vital cell nucleophiles that possess -SH or -NH_2_ groups, due to its α,β-unsaturated carbonyl structure [21,64]. Thus, ACR is oxidized to glycidamide, a reactive epoxide, which is conjugated to GSH [25]. Glycidamide also forms adducts with amino groups in DNA [26,27]. Increased lipid peroxidation levels occur as a result of GSH depletion to certain critical levels [25]. Therefore, by the reaction of ACR with GSH, S-conjugates of GSH are formed, which represent the first step in the biotransformation of electrophilic substances into mercapturic acids [27,29,49]. In the present study, reduction of hepatic GSH levels, and increase of lipid peroxidation, respectively, in the ACR-treated group compared to the control group, could be explained by the reaction between ACR and GSH. At the same time, with the administration of wines, it was observed that there was reduction of TBARS levels and increase of GSH values, due to the protective antioxidant effects of the constituent polyphenols. Thus, supplementation with both white and red wine of the diet of rats treated with ACR significantly reduced (*p* < 0.05) the hepatic level of TBARS in the 2 groups, WW + ACR and RW + ACR, compared to the PC group, treated only with ACR. These findings demonstrate that the oxidative lesions induced by ACR in the liver were ameliorated by treatment with both white and red wine, but especially red wine. Macedo et al. [63] also reported a reduction in hepatic TBARS level by supplementing the diet of rats with red wine with high antioxidant activity. Similar data were reported by Alturfan et al. [49], who induced oxidative stress with acrylamide in rats, and the protective effect was conferred by dietary supplementation with resveratrol.

Regarding hepatic GSH levels, a significant difference (*p* < 0.05) was observed between the different experimental groups, namely, the RW group compared to the control group, and the RW + ACR group compared to the PC group, suggesting that wine ingestion affected the homeostasis of the animals’ bodies. Increasing GSH levels in experimental groups given red wine might suggest an antioxidant response, even in the case of inducing oxidative stress with acrylamide. Similar results were reported by Alturfan et al. [49], while Gris et al. [65] did not obtain significant differences between the hepatic GSH levels of their different experimental groups, that were given eight different red wine samples, without inducing oxidative stress. In contrast to the results obtained for TBARS, where white wine supplementation conferred a protective effect against ACR, in the case of the hepatic GSH, the effect of white wine supplementation was weak, without significant increase in GSH level to provide protection. The favorable effects following the administration of wine, especially red wine, seem to be due to the presence of flavonoids and other phenolic compounds in wine, and are similar to those obtained in previous studies by Uzma et al. [57], where red wine relieved the hepatic oxidative stress induced by carbon tetrachloride in rats. The phenolic compounds present in wine may be responsible for the protective effects observed against ACR.

Antioxidant enzymes protect major molecules, such as lipids, proteins, and DNA from oxidative damage by inactivating oxidants. These antioxidant enzymes can act in a coordinated way to protect living tissues from oxidative damage [66]. Low levels of the enzymatic activities of CAT, SOD and GPx were recorded in the liver tissues of rats in the PC group treated with ACR compared to the control group, suggesting acute lesions caused by ACR. The harmful effects of ACR in the liver decreased with the administration of wines, with a change in the level of CAT and SOD to the normal level, depending on the type of wine administered.

Statistical analysis showed that the type of wine administered influenced the activity of SOD and CAT but did not show any significant effect on GPx. Thus, supplementing the diet with red wine in the RW + ACR group resulted in a significant increase (*p* < 0.05) in the enzymatic activity of SOD and CAT, compared to the PC group, while supplementing the diet with white wine did not produce significant differences in the activities of these enzymes. The positive effects of the red wine were more pronounced than those determined by the white wine, due to red wine having higher polyphenolic content compared to white wine. Rocha et al. [67] also obtained a significant increase in SOD activity when supplementing the diet of rats with resveratrol, while Uzma et al. [57] reported a significant increase in CAT activity in rats with oxidative stress induced by carbon tetrachloride due to dietary supplementation with red wine.

Regarding GPx activity, supplementing the diet with white or red wine did not produce significant differences between the groups treated with ACR, suggesting that in this case neither of the 2 types of wine was able to exert a protective effect against ACR. Similar results were reported by Macedo et al. [63], who did not obtain significant differences in GPx activity when supplementing the diet of rats with red wine with high antioxidant activity.

In general, when cells are exposed to eustress, they increase the activity and expression of antioxidant enzymes as a compensation mechanism to better protect them from free radical-induced damage by the activation of the nuclear factor erythroid 2-related factor 2/electrophile-responsive elements (Nrf2/EpRE) signaling pathway, which is a very important cytoprotective mechanism, ROS acting in low concentrations as cell mediators [68,69]. The reduction in the activities of antioxidant enzymes in groups of animals whose oxidative stress has been induced with ACR, might be due to the rapid consumption and depletion of these enzymes in combating free radicals generated during the development of oxidative stress. Given that ethanol can participate in free radical reactions, producing alkoxyl and hydroxyl radicals which increase cellular oxidative stress by producing O_2_^−^ and H_2_O_2_ [70,71], the association of alcohol with ACR could have generated a higher amount of O_2_^−^ in hepatocytes. In this case, the cells responded by modifying the activity of antioxidant enzymes, depending on the contribution of each phenolic compound and also on the synergism of the constituent compounds of the wines, to neutralize the excess ROS.

Due to the fact that the present study evaluated the effects of wine polyphenols, and taking into account the fact that alcohol is an enzyme inducer, all groups received the same concentration of alcohol, in the form of wine, that being 12.5% hydroalcoholic solution. Therefore, the differences in activity obtained between groups, namely the increase in the activity of antioxidant enzymes in animals from wine-treated groups, were attributed to the polyphenolic components of wines, rather than their ethanol content.

The main mechanisms of acrylamide hepatotoxicity are oxidative damage and mitochondrial dysfunction [72,73]. Regarding the first mechanism, oxidative stress, lipid peroxides and impaired antioxidant defense in the liver are linked to the activation of cytochrome P450 (CYP) 2E1, which functions as a central pathway in the formation of high levels of ROS, being also the only enzyme involved in biotransformation of ACR in glycidamide [53]. Therefore, CYP2E1-catalyzed ACR metabolism causes an imbalance between ROS production and elimination, in the sense of excessive ROS production, then results in lipid peroxidation and increases oxidative stress, which is related to liver damage [53,73,74]. Since ACR is not only a substrate, but also an inducer of CYP2E1, with CYP2E1 overexpression being induced by ACR intoxication and associated with increased oxidant production, the prevention and treatment of ACR-induced toxicity can be supported by antioxidants that have the ability to inhibit or to downregulate CYP2E1 [53]. Regarding the second mechanism of ACR hepatotoxicity, studies have confirmed the apoptotic property of ACR in a dose- and time-dependent manner in the liver, with long-term exposure to ACR causing mitochondrial collapse and leading to apoptosis [53,73,74]. Therefore, ACR treatment alters the potential of the mitochondrial membrane of hepatocytes and may alter the expression of nuclear factor-erythroid 2-related factor 2 (Nrf2) [74,75]. In addition, studies show that mitochondrial dysfunction causes the production of large amounts of ROS [75].

Polyphenols are known to be natural antioxidants with important antioxidant properties, which can significantly inhibit oxidative stress and inhibit the onset of mitochondrial dysfunction [74]. It is known that the mechanism of action of polyphenols is related to their ability to eliminate ROS, chelate metals, and influence the activity of enzymes, cell signal transduction pathways and gene expression [4,69]. When acting as direct antioxidants, polyphenols scavenge and neutralize free radicals involved in the etiology of various diseases, and also scavenge the 2,2-diphenyl-1-picrylhydrazyl radical used in the in vitro DPPH assay; thus preventing the oxidative damage caused by ROS and blocking the cascade of reactions in lipid peroxidation [69]. On the other hand, by modulating the nuclear factor erythroid 2-related factor 2/electrophile-responsive elements (Nrf2/EpRE) signaling pathway, polyphenols increase the activity of some antioxidant and detoxifying enzymatic systems and down-regulate the Nuclear Factor kappa B (NF-кB) system [69]. As polyphenols have a very complex antioxidant activity, it is important that the results support its exercise through several mechanisms of action. Therefore, through the markers used both in vitro (DPPH) and in vivo (SOD, CAT, GPx), wine polyphenols were able to demonstrate their protective capacity exerted by several mechanisms, mentioned above.

Thus, flavonoids, the main polyphenols contained in wine, are known to prevent lipid peroxidation and low-density lipoprotein oxidative changes due to their antioxidant properties [4,6]. They have antioxidant action through electron transfer and formation of electrophilic metabolites, while ortho- or para-dihydroxyphenols can be oxidized to quinones which act by increasing Nrf2 [69]. Considering the flavonoid-rich content (flavan-3-ols, such as catechin, epicatechin gallate, gallocatechin, but also flavonols, such as derivatives of myricetin, quercetin, isorhamnetin, laricitrin, syringetin and kaempferol) of the wines tested in this study [39], the results obtained support a positive association between the intake of wine flavonoids and the reduction of oxidative stress and its consequences.

Along with flavonoids, the anthocyanins (derivatives of cyanidin, petunidin, delfinidin, peonidin and malvidin) contained in the red wine tested in the study [39] contribute to the reduction of lipid peroxidation, resulting from oxidative stress, as reported by Kolota et al. [4]. The red wine fraction containing anthocyanins has been shown to be the most effective in its ability to trap ROS and inhibit LDL oxidation, compared to two other fractions containing phenolic acids, flavonols, procyanidins and catechins. The properties of anthocyanins are due to their peculiar chemical structure, being very reactive towards ROS because of their electron deficiency [76]. In addition, anthocyanins have shown cellular antioxidant mechanisms comparable to, or greater than, other micronutrients, such as vitamin E [76]. Regarding the ability of flavonoids and anthocyanins to prevent lipid peroxidation, a study in a human population supports a positive association between regular consumption of red wine and a reduction in serum MDA levels, demonstrating that lipid peroxidation was reduced following a regular consumption of 100 mL of red wine/day, through a mechanism involving the intestinal microbiota [6].

The antioxidant activity demonstrated in vivo by the tested wines was also due to their stilbenes content, particularly of resveratrol, the main representative, which have capacity to inhibit the so-called “oxidative burst” (production of O_2_^−^ and H_2_O_2_), to regulate CAT, SOD, GPx, glutathione reductase, GST activities, as well as to induce endogenous antioxidant defenses, such as the Nrf2 pathway [69,77].

In summary, the results of recent research given in the literature show that ACR toxicity is due to oxidative stress induced by ROS generation and activation of the transcription factor NF-κB, involved in the generation of the inflammatory response by the release of cytokines, including IL-6, TNF-α, and IL-1β [21,78]. Furthermore, a number of studies have confirmed this hypothesis by the fact that various antioxidant compounds, such as sulforaphane [79], blueberry anthocyanins [80], or N-acetylcysteine [81], have the ability to reverse the toxicity of ACR, by activating the transcription factor Nrf2 and its Nrf2/ARE signaling pathway, and, consequently, inhibit NF-κB. It is known that several wine polyphenols, such as resveratrol, catechins, quercetin, ellagic acid and ellagitannins, gallic acid and gallotannins, etc., have significant antioxidant activity, exerted by several mechanisms of action, including activation of Nrf2 and inhibition of NF-κB [69]. Indeed, previous phytochemical analysis of the wines we tested in this study showed that they have a high content of such polyphenols, and the results obtained in vivo show that these wine samples administered to rats decrease ACR toxicity by reducing oxidative stress.

## 5. Conclusions

In conclusion, the administration of ACR (250 µg/kg body weight over a period of 28 days) induces hepatotoxicity in rats, by altering MDA, TBARS and GSH levels, antioxidant enzyme activities, liver enzyme activities and by producing hepatic histopathological changes. Our results showed that wine polyphenols increased GSH content, normalized the activities of the antioxidant enzymes CAT and SOD, inhibited lipid peroxidation in the liver, improved the AST level and attenuated morphological changes, especially in the case of red wine. Therefore, the antioxidant properties of wine polyphenols may be considered to be primary mechanisms in protection against ACR-induced oxidative stress and toxicity.

## Figures and Tables

**Table 1:** Total phenolic content (TPC), DPPH radical scavenging activity (%) and total antioxidant activity (TAA) of the two wine samples used in this study (mean value (*n* = 3)).

White Wine Sample	TPC (mg GAE/L)	DPPH Radical Scavenging Activity (%)	TAA (mM TE/L)
FR_Jid2011_	245 *	51 *	0.93 *
Red Wine Sample	TPC (mg GAE/L)	DPPH Radical Scavenging Activity (%)	TAA (mM TE/L)
FN_Toh2010_	2359 *	95 *	9.84 *

GAE: Gallic acid equivalents; TE: Trolox equivalents. * Data from Banc et al. [39].

**Table 2:** Experimental design.

Group	Number of Animals	Intragastric Gavage	Diet
Control (C)	10	12.5% (*v*/*v*) hydroalcoholic solution	standard
Positive control (PC)	10	12.5% (*v*/*v*) hydroalcoholic solution + acrylamide 250 µg/kg of weight, 1% (*m*/*v*) aqueous solution	standard
White wine (WW)	10	FR_Jid2011_ white wine	standard
White wine + acrylamide (WW + ACR)	10	FR_Jid2011_ white wine + acrylamide 250 µg/kg of weight, 1% (*m*/*v*) aqueous solution	standard
Red wine (RW)	10	FN_Toh2010_ red wine	standard
Red wine + acrylamide (RW + ACR)	10	FN_Toh2010_ red wine+ acrylamide 250 µg/kg of weight, 1% (*m*/*v*) aqueous solution	standard

**Table 3:** Effects of white/red wine on absolute liver weight and relative liver weight (% of body weight) of rats in the 6 experimental groups at the end of the experiment.

	Experimental Groups					
	C	PC	WW	WW + ACR	RW	RW + ACR
Absolute liver weight (g)	4.78 ± 0.15 ^a^	6.04 ± 0.17 ^b^	4.66 ± 0.18 ^a^	6.02 ± 0.15 ^b^	4.79 ± 0.17 ^a^	5.99 ± 0.03 ^b^
Relative liver weight (%)	2.58 ± 0.08 ^a^	3.15 ± 0.09 ^b^	2.39 ± 0.09 ^a^	2.94 ± 0.08 ^b^	2.46 ± 0.09 ^a^	2.82 ± 0.02 ^a,b^

Values are expressed as mean ± SEM (*n* = 10). ^a,b^ Mean values not sharing the same superscript letter within a row are different at *p* < 0.05. C−hydroalcoholic solution group; PC−hydroalcoholic solution + acrylamide group; WW−white wine group; WW + ACR−white wine + acrylamide group; RW−red wine group; RW + ACR−red wine + acrylamide group.

**Table 4:** Effects of white/red wine on aspartate aminotransferase and alanine aminotransferase levels in the plasma of rats from the 6 experimental groups.

	Experimental Groups					
	C	PC	WW	WW + ACR	RW	RW + ACR
AST (U/mL plasma)	101.29 ± 4.96	162.06 ± 23.15 ^a^	116.31 ± 15.06	130 ± 12.29	105.17 ± 6.45 ^b^	108.3 ± 10.06 ^b^
ALT (U/mL plasma)	60.34 ± 9.71	88.93 ± 10.27 ^a^	52.93 ± 3.83 ^b^	70.77 ± 14.79	44.94 ± 5.56 ^b^	62.68 ± 11.61

Values are expressed as mean ± SEM (*n* = 10). ^a^ There are significant differences when compared to the control group (C) at the significance level *p* < 0.05. ^b^ There are significant differences when compared to the positive control group (PC) at the significance level *p* < 0.05. AST−aspartate aminotransferase; ALT−alanine aminotransferase. C−hydroalcoholic solution group; PC−hydroalcoholic solution + acrylamide group; WW−white wine group; WW + ACR−white wine + acrylamide group; RW−red wine group; RW + ACR−red wine + acrylamide group.

## Data Availability

The data presented in this study are available in this manuscript.

## Supplementary Materials

The following supporting information can be downloaded at: https://www.mdpi.com/article/10.3390/antiox11071347/s1, Figure S1: Chromatogram of a rat plasma sample from RW group for determination of MDA; Figure S2: Chromatogram of a rat plasma sample from PC group for determination of total GSH; Figure S3: Chromatogram of a rat plasma sample from RW + ACR group for determination of reduced GSH; Table S1: The average body weight of rats from the 6 experimental groups at the beginning and at the end of the 28 experimental days.
