# Supplementary material for: Protective Effects of Wine Polyphenols on Oxidative Stress and Hepatotoxicity Induced by Acrylamide in Rats
Source: Antioxidants (Basel). 2022 Jul 10;11(7):1347. doi: 10.3390/antiox11071347 (PMC9312107; doi:10.3390/antiox11071347)
Supplement: Supplementary file 1 [file antioxidants-11-01347-s001.zip › antioxidants-1789000-supplementary.pdf]

antioxidants

MDPI

Article

Supplementary Materials

1. Figures

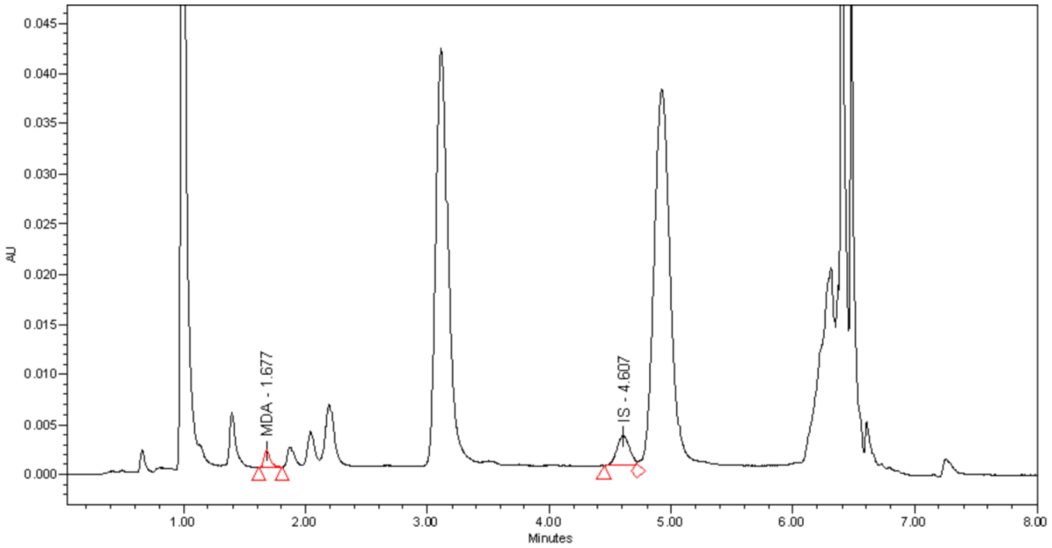

Figure S1. Chromatogram of a rat plasma sample from RW group for determination of MDA. RW – red wine group ($FN_{Toh2010}$ red wine (7 mL/kg/day)). IS – internal standard.

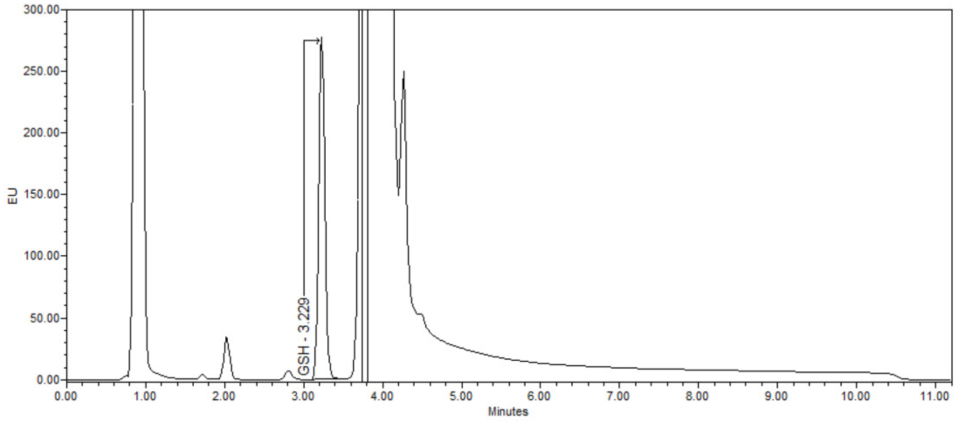

Figure S2. Chromatogram of a rat plasma sample from PC group for determination of total GSH. PC – hydroalcoholic solution + acrylamide group (12.5% (*v*/*v*) hydroalcoholic solution (7 mL/kg/day) + acrylamide (250 µg/kg of weight, 1% (*m*/*v*) aqueous solution)).

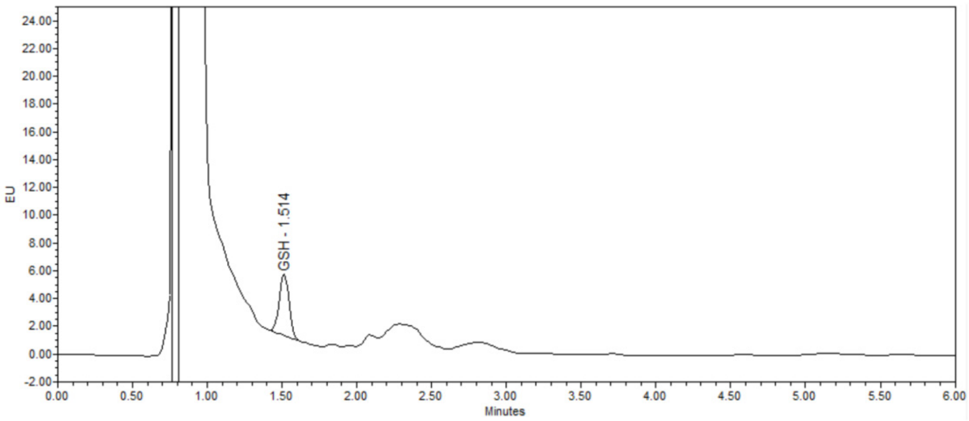

Figure S3. Chromatogram of a rat plasma sample from RW + ACR group for determination of reduced GSH. RW + ACR − red wine + acrylamide group ($FN_{Toh2010}$ red wine (7 mL/kg/day) + acrylamide (250 µg/kg of weight, 1% (*m*/*v*) aqueous solution)).

2. Tables

Table S1. The average body weight of rats from the 6 experimental groups at the beginning and at the end of the 28 experimental days.

	Experimental groups					
	**C**	**PC**	**WW**	**WW + ACR**	**RW**	**RW + ACR**
Initial body weight (g)	140.60 ± 1.80^{a}	149.90 ± 2.13^{b}	142.50 ± 3.01^{a}	157.11 ± 3.80^{b}	144.00 ± 3.16^{a}	167.70±5.77^{b}
Final body weight (g)	185.40 ±4.46^{a}	191.90 ± 5.24^{a}	195.0 ± 5.74^{a}	204.89 ± 4.09^{a}	194.80 ± 6.22^{a}	212.50 ±4.09^{b}

Values are expressed as mean ± SEM (*n* = 10). ^{a,b} Mean values not sharing the same superscript letter within a row are different at $p < 0.05$. C − hydroalcoholic solution group; PC − hydroalcoholic solution + acrylamide group; WW − white wine group; WW + ACR − white wine + acrylamide group; RW − red wine group; RW + ACR − red wine + acrylamide group.
